# Computed tomography assessment after out-of-hospital cardiac arrest resuscitation

**DOI:** 10.1016/j.resplu.2026.101346

**Published:** 2026-04-24

**Authors:** Saleem M. Halablab, Frances Shofer, Aarthi Kaviyarasu, Oscar J.L. Mitchell, William Reis, Taylor Brothers, David Fischer, Benjamin S. Abella, John Greenwood

**Affiliations:** a Department of Emergency Medicine, Center for Resuscitation Science, Perelman School of Medicine at the University of Pennsylvania, Philadelphia, PA, USA; b Department of Anesthesiology and Critical Care, Perelman School of Medicine at the University of Pennsylvania, Philadelphia, PA, USA; c Department of Epidemiology & Biostatistics, Department of Emergency Medicine Hospital of the University of Pennsylvania, Philadelphia, PA, USA; d Division of Pulmonary, Allergy, & Critical Care, Perelman School of Medicine, University of Pennsylvania, Philadelphia, PA, USA; e Division of Neurocritical Care, Department of Neurology, Perelman School of Medicine at the University of Pennsylvania, Philadelphia, PA, USA; f Department of Emergency Medicine, Icahn School of Medicine at Mount Sinai, New York, NY, USA

**Keywords:** Out of hospital cardiac arrest, Computed tomography, Resuscitation, Pulmonary embolism, Intracranial hemorrhage, Pneumonia

## Abstract

**Aims:**

The present study aims to assess the incidence of causes of cardiac arrest and sequelae after resuscitation from OHCA (out-of-hospital cardiac arrest) identified on early, post-arrest Computed Tomography (CT).

**Methods:**

This is an observational, registry study of adult OHCA patients admitted to three hospitals at the University of Pennsylvania Health System who achieved sustained return of spontaneous circulation between January 2019 and October 2020. Data collected encompassed demographics, arrest characteristics, basic metabolic profiles, CT imaging data, and clinical care notes. Primary outcome is survival to hospital discharge and secondary outcomes included neurologic outcome, CT findings and interventions, and IV contrast and kidney injury rates.

**Results:**

Out of 161 patients, 117 received at least one CT in the ED after return of spontaneous circulation. The most common pathologies identified by post-arrest CT scans include cerebral oedema (30/117); pulmonary embolism (8/87); sternal fractures (9/87); pulmonary infiltrates (69/87) that included aspiration, pneumonia, or pneumonitis; deep venous thrombosis (4/64); pulmonary contusions (4/87); hematomas (2/87); flail chest (1/87).

**Conclusion:**

Early CT imaging after ROSC from OHCA regularly identified potential causes of cardiac arrest and important sequelae of resuscitation efforts. Implementing larger scale prospective studies comparing such protocols and standard-of-care is important to determine impact on OHCA outcomes.

## Introduction

Post-resuscitation care for patients after out-of-hospital cardiac arrest (OHCA) remains a major clinical challenge. According to national data from the Cardiac Arrest Registry to Enhance Survival, there are approximately 1000 OHCA patients are managed by emergency services daily across the United States, with survival to hospital admission and hospital discharge are 24% and 9%, respectively.^1^, ^2^ Diagnostic testing after sustained return of spontaneous circulation (ROSC) differs widely between institutions due to differences in available resources and the diverse aetiologies of OHCA, which necessitate tailored diagnostic and therapeutic interventions. Timely identification of reversible causes or complications of OHCA are critical for successful post-resuscitation care, highlighting the importance of standardized diagnostic evaluations in the Emergency Department (ED).

Following ROSC, patients typically undergo various testing, including an immediate electrocardiogram (ECG) and blood tests, to evaluate organ function and biomarker analysis. Additional testing is based upon clinical evaluation.^3^ Several studies have examined the role of diagnostic tools in post-resuscitation care such as electrocardiography, ultrasound, coronary angiography, and various biomarkers.^4^, ^5^ While these studies are integral to improving post-arrest care, and recent guidelines recommending considering various diagnostic imaging in post-ROSC care,^6^, ^7^ further research is necessary to support the use of diagnostic imaging in post-ROSC care.

Computed tomography (CT) is a valuable tool available in most EDs that regularly helps guide clinical decisions by emergency physicians and care teams. Whole-body or pan-CT scan protocols have been found to be valuable in evaluating acute critical illness, particularly trauma, where it facilitates the timely detection of injuries and emergent surgical planning.^8^, ^9^, ^10^ In OHCA, CT has been shown to play an important role in post-arrest care in several studies. Recent reports found that CT imaging is safe, expedites diagnosis of potential OHCA causes, and influences clinical care.^11^, ^12^, ^13^ Several observational studies highlight the ability of CT acquisition to detect potential causes or sequelae to cardiac arrest management such as pneumonia or aspiration, pneumothorax, rib or sternal fractures, haematomas, bowel ischaemia, visceral organ lacerations, and many others conditions, at varying rates.^14^, ^15^, ^16^, ^17^

A recent analysis of a single-hospital prospective study, with a retrospective control group, employed a standardized head-to-pelvis sudden death CT protocol during post-resuscitation care for idiopathic OHCA. This CT protocol exhibited improved diagnostic yield of causes of OHCA when compared to non-protocolized post-arrest evaluation.^11^ While these findings highlight CT's potential, more data on current institutional practices and its diagnostic accuracy are needed to confirm these results and guide its integration into post-arrest care guidelines. The objective of this study aims is to evaluate the role of CT imaging in early post-arrest care using a multi-center OHCA database.

## Methods

### Study design and setting

This is an observational study, performed using data from the Penn Center for Resuscitation Science OHCA registry. This study was approved by the Penn Institutional Review Board (IRB #849677). Consecutive OHCA patients who presented to three University of Pennsylvania Health System EDs, including Hospital of the University of Pennsylvania, Penn Presbyterian Medical Center, and Pennsylvania Hospital from over an 18-month period (January 2019–October 2020) were included in this study. All registry data was manually collected from patient electronic medical records and emergency medical services records if available. All data were recorded on a secure and standardized case report form in Research Electronic Data Capture (REDCap) and was reviewed by the principal investigators for validity and quality.^18^, ^19^

### Study population

All patients greater than 18 years-of-age who achieved sustained ROSC for at least 20 mins while in the emergency department were included in this study. We excluded patients if they were transferred from outside hospitals for post arrest care due to incomplete data records.

### Variables and outcomes

The primary outcome for this study is survival to hospital discharge between patients who received a CT scan in the Emergency Department and those who did not. Secondary outcomes included neurologic outcome, the incidence of potential causes and/or sequelae of OHCA resuscitation identified on ED CT imaging, intervention(s) performed based on CT findings, and the association between intravenous contrast exposure and AKI.

Patient demographics included age, sex, race, and ethnicity. Arrest characteristics such as initial rhythm, arrest location, presence/absence of a witness, and bystander CPR or AED use were included. During this time interval, the University of Pennsylvania did not have a protocolized approach to ordering post-arrest CT scans. We collected data on all CT imaging performed in the ED for the OHCA patients including time from ED arrival to CT scan, use of intravenous contrast, and imaging results. Given the retrospective nature of the study, decision to pursue CT imaging was made by primary ED clinical care teams based solely on clinical judgement. All CT imaging radiology reports for the included patients were reviewed and the findings were then grouped into specific predetermined categories in REDCap, encompassing potential causes or sequelae of the arrest. Clinical care notes were abstracted and analyzed to assess for the impact of CT findings on medical decision making. Safety events were defined as any episode of persistent change in haemodynamics, hypoxia, or need to discontinue a CT scan prior to completion. Acute kidney injury (AKI) was assessed using the Kidney Disease: Improving Global Outcomes (KDIGO) definition of AKI.^20^ We collected the first 3 creatinine measurements for all included patients and then excluded those with end-stage renal disease or on dialysis and those with less than 2 measurements of Creatinine during hospitalization. We then identified AKI in those with an increase in creatinine by 0.3 or more than 1.5 times their baseline creatinine values (KDIGO definition) and compared it to exposure to CT with contrast.

### Statistical analysis

Descriptive summary statistics were used to characterize the study population, using mean ± standard deviation or median and interquartile range for continuous variables, such as age, and frequencies and percentages for categorical variables such as gender, race, and initial rhythm. To assess differences in survival, outcome, and arrest characteristics, based on CT utilization, Fisher’s exact tests or Wilcoxon rank sum tests were performed. All analyses were performed using SAS statistical software (version 9.4, SAS Institute (Cary NC)). A *p*-value < 0.05 was considered significantly different.

## Results

### Characteristics of study subjects

**Fig. 1:**
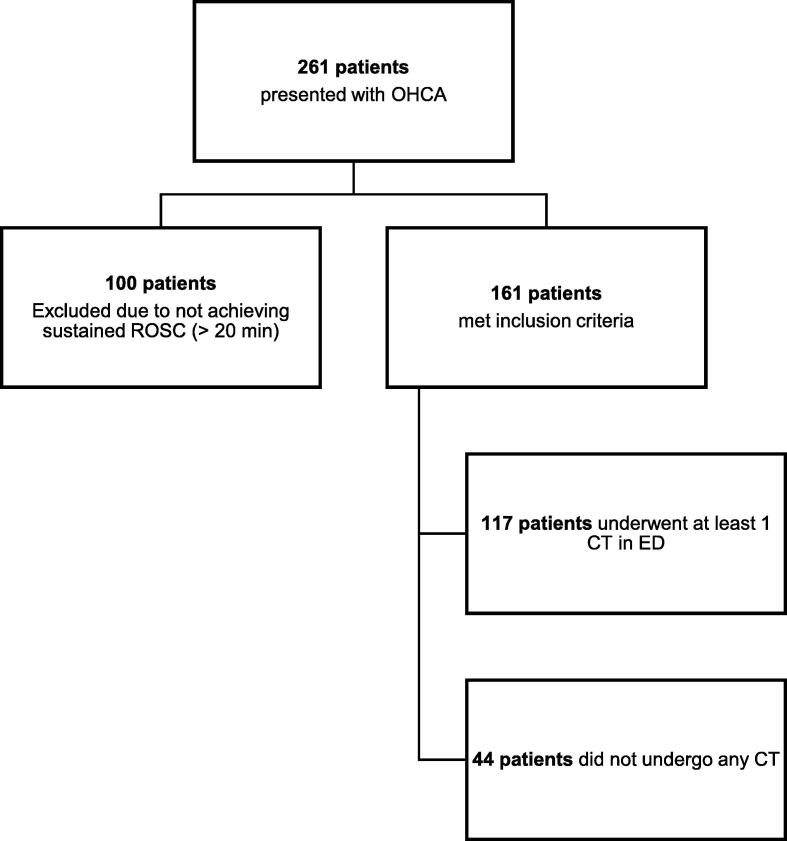
**STROBE diagram illustrating the study population**. OHCA, out-of-hospital cardiac arrest; ROSC, sustained return of spontaneous circulation; CT, computed tomography.

A total of 261 patients were eligible for inclusion during the defined study period of January 2019 and October 2020. Of those, 161 achieved sustained ROSC, of which, 117 patients (72.7%) underwent at least one form of CT imaging in the ED (see Fig. 1), including 117 head CTs, 87 chest CTs, and 64 abdomen/pelvis CTs. Median time to any first CT was 2.0 h [IQR: 1.4, 2.8]. There was no difference in patient demographics and arrest characteristics between patients that did and did not receive a CT examination in the ED (Table 1).

**Table 1:** Demographic and arrest characteristics of OHCA patients that underwent at least one CT in the ED and those that did not.

**Demographic/arrest characteristic**		**ED CT (*n* = 117)**		**None (*n* = 44)**		***p*-value**
		***n***	**(%)**	***n***	**(%)**	
Age (mean ± SD)		60.5 ± 16.1		61.5 ± 14.4		0.71
Gender	Male	62	(53.0)	27	(61.4)	0.25
	Female	53	(45.3)	15	(34.1)	
	Other	2	(1.7)	2	(4.6)	
Race	White/Caucasian	29	(26.1)	12	(29.3)	0.94
	Black/African American	78	(70.3)	28	(68.3)	
	Asian/Pacific Islander	4	(3.6)	1	(2.4)	
Witnessed arrest		80	(70.8)	32	(76.2)	0.43
Bystander CPR		39	(35.8)	23	(56.1)	0.064
Initial rhythm	Non-shockable	16	(13.9)	9	(20.9)	0.33
	Shockable	99	(86.1)	34	(79.1)	

CT, computed tomography; ED, emergency department; OHCA, out-of-hospital cardiac arrest; CPR, cardiopulmonary resuscitation.

### Main results

Patients that underwent at least one form of CT were more likely to be admitted alive (98% vs. 88.6%, *p* = 0.017), but there were no significant differences in survival to discharge (23.7% vs 33.3%, *p* = 0.291), at 3 months (13.4% vs. 22%, *p* = 0.215), 6 months (12.5% vs. 18%, *p* = 0.425), or command-following recovery (*p* = 0.492) between patients who underwent any CT examination in the ED and those that did not (Table 1, Table 2). Furthermore, a significantly higher proportion of patients that did not undergo CT were diagnosed with an ST-segment elevation myocardial infarction (STEMI) in the ED (9.7% vs 29.3%, *p* = 0.004).

**Table 2:** Comparison of ED CT and no ED CT groups.

		**ED CT (*n* = 117)**		**None (*n* = 44)**		***p*-value**
		***n***	**(%)**	***n***	**(%)**	
Admitted alive		115	(98.3)	39	(88.6)	0.02
Discharged alive		27	(23.7)	13	(33.3)	0.29
Survival to 3-months after discharge		15	(13.4)	9	(22)	0.22
Survival to 6-months after discharge		14	(12.5)	7	(18)	0.43
STEMI		11	(9.7)	12	(29.3)	0.004
Length of hospital stay (median [IQR])		5	[1–12]	3	[1–6.5]	0.06
Command-following	On arrival	2	(1.9)	2	(5.1)	0.49
	Recovered later	17	(15.7)	5	(12.8)	
	Never recovered	89	(82.4)	32	(82)	

CT, computed tomography; ED, emergency department; OHCA, out-of-hospital cardiac arrest; STEMI, ST-segment elevation myocardial infarction.

Potential etiology of cardiac arrest diagnosed by CT scan were as follows: Intracranial hemorrhage in 5/117 patients (4.3%) on head CT, 3 of which received medical conservative therapy to decrease intracranial pressure with head of bed elevation, hypertonic saline or mannitol, and transient hyperventilation. Pulmonary embolism was identified in 8/87 (9.2%) patients on chest CT, 7 of which were acute and treated with thrombolytics, anticoagulants, and/or mechanical thrombectomy. Deep venous thrombosis was detected in 3/64 patients (4.7%) on abdomen and pelvis CT, 2 of whom received anticoagulation therapy. A total of 44 patients did not undergo any CT imaging in the ED, of which 39 were admitted alive. Of these patients, 12 patients had STEMI. Out of the remaining 29 patients, 12 underwent a CT after hospital admission.

Suspected sequelae of post-arrest resuscitation found on CT imaging included cerebral oedema 30/117 patients (25.6%) on head CT, 10 of which received medical treatment. Pulmonary infiltrates concerning for aspiration, pneumonia, or pneumonitis, or contusions were found on 69/87 (79.3%) patients on chest CT, 30 of whom received antibiotic treatment. Rib fractures were found in 47/87 (54%) patients, sternal fractures in 9/87 (10.3%) patients, sternal hematomas in 2/87 (2.3%) patients, pneumothorax in 9/87 (10.3%), and flail chest in 1/87 (1.2%) patient. A summary of all findings on different CT modalities can be found in Table 3.

**Table 3:** Summary of CT findings by imaging modality.

	**Finding**	**Head CT**	**Chest CT**	**Abdomen pelvis CT**
Potential causes of OHCA	Intracranial hemorrhage	5 (4.3%)		
	Pulmonary embolism		8 (9.2%)	
	Deep venous thrombosis			3 (4.7%)

Sequelae of resuscitation	Cerebral oedema	30 (25.6%)		
	Pulmonary infiltrates		69 (79.3%)	
	Rib fractures		47 (54%)	
	Sternal fractures		9 (10.3%)	
	Sternal hematoma		2 (2.3%)	
	Pneumothorax		9 (10.3%)	
	Flail chest		1 (1.2%)	

CT, computed tomography; OHCA, out-of-hospital cardiac arrest.

A total of 67 patients received intravenous contrast for CT. Out of 145 patients with available renal function data at 48 h, we found no significant difference in AKI rates between those who received IV contrast and those that did not (20.3% vs. 19.6%, *p* = 0.93). We also did not identify any safety events that led to an incomplete scan or need to discontinue ordered CT scans.

## Discussion

This multicentre, observational database study did not find an association between early ED diagnostic CT scan and mortality after OHCA. Early CT scans frequently identified potential aetiologies of cardiac arrest, as well as potential sequelae of post-arrest resuscitation. Our results highlight that CT is a safe modality that is able to detect significant and critical findings that influence clinical care for OHCA patients after successful resuscitation. Furthermore, our results indicate that CT imaging is highly utilized but not uniformly performed in real-world practice for the assessment of patients after OHCA resuscitation.

Similar to the recent CT first study, and other retrospective studies, we did not find any significant difference in survival to hospital discharge or neurologic outcome in patients who received at least one CT and those who did not receive a post-arrest CT scan.^8^, ^14^, ^15^, ^16^, ^21^ It does not appear that the baseline clinical and arrest differences between the scanned and non-scanned populations affected patient outcome, but some clinical not collected, including time to first CPR, total down time (time from arrest to ROSC), presence or absence of a witness, and transport time. Electrocardiography remains one of the gold standard tests in post-OHCA care, as it can identify patients with a primary cardiac cause, who have been found to have a lower in-hospital mortality.^22^ Where early CT scanning does appear to hold promise, is in the rapid diagnosis of non-cardiac causes of OHCA as well as other high-risk sequela of resuscitation care.^23^, ^24^

In this study, early CT imaging identified a number of potential causes and sequelae of cardiac arrest resuscitation. Cerebral oedema or hemorrhage is common after cardiac arrest. In this study, cerebral oedema and was identified in 25.6% of head CT and intracranial hemorrhage in 4.3% of head CT, which is consistent with previous literature.^25^, ^26^ Post-arrest brain injury is one of the most common causes of morbidity and mortality after cardiac arrest. Updated post-resuscitation care guidelines from major societies in 2025 started recommending routine brain imaging after cardiac arrest.^6^, ^7^ In response to these evolving data, our institution has recently developed a standardized approach to post-arrest neuroprognostication which includes routine early head CT and MRI imaging.^27^ Pulmonary and thoracic pathology, including signs of early acute respiratory distress syndrome (ARDS), were identified in over 80% of patients who received a chest CT in the ED and was the most common pathology identified. Previous literature has reported that ARDS can occur in 48–71% of intubated post arrest patients and is associated with increased mortality.^28^, ^29^ Initial chest radiography would likely identify many patients with early ARDS but compared to chest CT has a significant inter-observer variability and a much lower sensitivity.^30^, ^31^ Identification of early radiographic evidence of ARDS with cross-sectional imaging could increase the likelihood of prescribing lung protective ventilation and preventing secondary lung injury. Lastly, pulmonary embolism was found in almost 10% of OHCA survivors, which requires nuanced therapeutic decisions regarding anticoagulation and additional post-arrest care.

Our results also highlight the clinical variability in post-arrest imaging. Most patients received a head CT at our institution, but the frequency of chest, abdominal, and pelvic CT scans were much less common. It is unclear if a targeted vs. broad approach to post-arrest imaging can improve patient-centered outcomes, but current literature suggests that early protocolized CT scanning can reduce time to identify critical diagnoses. The Branch et al team have demonstrated through a series of studies that CT scanning is an effective, safe, and quick procedure that expedites diagnosis after OHCA resuscitation.^21^ This, combined with recent observational data outlining the diagnostic abilities of CT after OHCA, may motivate the routine use of post-ROSC protocolized CT scanning. Our data reinforces these findings that early post-arrest CT scans are safe and reduce time to critical diagnoses. Additional large-scale studies are needed to clearly define the utility and survival benefit of a standardized CT protocol.

### Limitations

This study has several limitations that future clinical research could address. First, as an observational database study, it is limited by the accuracy and completeness of electronic medical record documentation. Intervention rates are likely underreported, but we attempted to maximize accuracy by thoroughly reviewing clinical documentation and applying standardized definitions before data collection and intervention classification. Additionally, CT utilization may have been lower than expected due to clinical considerations such as safety, appropriateness, and time constraints. This could introduce selection bias, potentially affecting the incidence of observed findings. In addition, patients undergoing ED CT may have been less sick than those that did not undergo CT in the ED, highlighting the limitation of interpretation of the neurologic and survival outcome differences between both groups due to the introduced selection bias. Our institution's protocol during the study period encouraged early head CT in OHCA patients, which may explain the high rate of post-arrest head CTs. Additionally, when comparing the CT vs non-CT groups, an immortal time bias may present as some patients may have been too unstable to undergo CT imaging in the ED. Furthermore, many of the patients included in this study died within a short time after admission, limiting the identification of AKI in relation to CT contrast exposure.

## Conclusions

These study results confirm and extend the conclusions of previous studies that emphasize the value of early, standardized CT imaging in post-resuscitation care for OHCA patients. We believe that this would motivate implementing larger scale prospective studies aimed at comparing standard-of-care to a protocolized post-ROSC CT examination to further validate these results through a real-world assessment.

## CRediT authorship contribution statement

**Saleem M. Halablab:** Writing – original draft, Project administration, Methodology, Formal analysis, Data curation, Conceptualization. **Frances Shofer:** Software, Resources, Methodology, Formal analysis, Data curation. **Aarthi Kaviyarasu:** Writing – original draft, Project administration, Data curation. **Oscar J.L. Mitchell:** Writing – review & editing, Supervision, Resources, Investigation. **William Reis:** Project administration, Data curation. **Taylor Brothers:** Project administration, Data curation. **David Fischer:** Writing – review & editing, Supervision, Resources, Investigation. **Benjamin S. Abella:** Writing – review & editing, Writing – original draft, Supervision, Resources, Methodology, Conceptualization. **John Greenwood:** Writing – review & editing, Writing – original draft, Supervision, Resources, Investigation, Formal analysis.

## Declaration of competing interest

The authors declare that they have no known competing financial interests or personal relationships that could have appeared to influence the work reported in this paper.
